# Use of Acupuncture for the Treatment of Sports-Related Injuries in Athletes: A Systematic Review of Case Reports

**DOI:** 10.3390/ijerph17218226

**Published:** 2020-11-06

**Authors:** Ji-Won Lee, Jun-Hwan Lee, Song-Yi Kim

**Affiliations:** 1 College of Korean Medicine, Gachon University, Seongnam 13120, Korea; behelia11@gmail.com; 2 Clinical Medicine Division, Korea Institute of Oriental Medicine, Daejeon 34054, Korea; 3 Korean Medicine Life Science, University of Science & Technology (UST), Campus of Korea Institute of Oriental Medicine, Daejeon 34054, Korea

**Keywords:** sports injury, athlete, acupuncture, case report, case series, review

## Abstract

Acupuncture is one of the representative complementary and alternative medicine treatments used for various types of pain. This systematic review summarized and analyzed clinical case reports/series utilizing acupuncture for treating sports injuries in athletes, thereby providing the basis for further research to establish clinical evidence on acupuncture treatment in sports medicine. A comprehensive literature search was conducted in Embase including MEDLINE up to 21 August 2019 without language and publication date restrictions. Due to the heterogeneity of each study, explanatory and descriptive analyses were performed. As a result, in each case report/series, it was confirmed that acupuncture was applied for treating various types of sports injuries experienced by athletes. Acupuncture can help relieve short-term pain and recover from dysfunction and has been used as a useful, noninvasive, and conservative modality for managing sports injuries such as lateral meniscus rupture, femoral acetabular impingement, ganglion cysts, and sports hernia. In addition, acupuncture has been suggested as a treatment worth trying for diseases such as yips and delayed onset muscle soreness. The included cases showed some potential of acupuncture in the treatment of various types of sports injuries, beyond pain control in musculoskeletal disorders. However, considering that this review was based on case reports/series, a limited understanding of the clinical value of acupuncture in athletes is required. In the future, more specific research questions and hypotheses should be addressed to generate evidence based on experimental research.

## 1. Introduction

In recent years, exercise has served a multitude of functions, in addition to promoting health [1]. Regular exercise not only improves an individual’s health, but is also considered as a “therapy” for people from all walks of life, such as the pediatric and elderly population, those with chronic diseases, or those with obesity [2,3]. Exercise generally has obvious benefits, but it also requires proper techniques, strength, and frequency [1]. At present, as the population of sports enthusiasts increases, sports injuries have become a prevalent affliction [4]. Musculoskeletal disorders account for most sports injuries and tend to recur frequently [5,6]. Although rare compared to musculoskeletal diseases, sports medicine encompasses various kinds of diseases that preclude sports activities, including psychological issues [7].

In comparison to recreational athletes or non-athletes, elite athletes are deemed to possess more specific goals and are more motivated for improving performance [8]. It is natural that sports injuries are very crucial to elite athletes because they directly affect their careers and economic profit. Therefore, an athlete-centered, long-term, return-to-play (RTP) strategy for those with sports injuries is deemed to be of utmost priority [9]. The objective of an athlete’s treatment and rehabilitation is to return to their respective sports with minimal performance loss, whereas that of non-athletes is to return to normal daily activities [9]. Accordingly, athletes’ sports injuries often lead to complicated considerations in the doctors’ decision-making process [10].

Regarding therapeutic approach for sports injuries, it is important to consider the patient background, including the type of sport and nature of the injury (acute or overuse), or their usual training or match schedules, as well as whether they belong to professional or recreational sports [11]. Thus, practitioners need to know the wealth of clinical evidence about sports-specific profiles and various treatment options [12]. Experimental studies, including randomized controlled trials (RCTs) or controlled clinical trials (CCTs), commonly provide valuable evidence [13] but may have limitations, especially in athletes. Although little has been studied about the motivations of elite athletes to participate in clinical studies, the benefits, generally considered being patients’ motivations in clinical study, including access to health care and personal benefit, altruism, and monetary incentives [14], may not be attractive to elite athletes. This could be the reason why experiments conducted on athletes were mainly uncontrolled trials involving the participation of an entire team or experiments conducted with young nonprofessional athletes or in a heterogeneous group of athletes. Another limitation is that the research inclusion criteria required by RCTs are often too restrictive for a wide range of sports injuries, and the statistical results of RCTs can often be regarded as ignoring or equalizing the participants’ background characteristics [13]. A case report is a detailed report of an individual patient’s diagnosis, treatment, response to treatment, and follow-up, while a case series aggregates individual cases who received similar treatments into one report [15]. Essentially, clinical case reports or series (case reports/series) not only belong at the bottom of the hierarchy of evidence-based medicine (EBM), but also provide a lower causal relationship compared to RCTs [16]. However, they can provide important and beneficial clues about potential treatments for athletes. In addition, case reports/series are the primary sources of information on new treatment options, responses to treatment, details of management, long-term follow-up, and unexpected adverse events (AEs) of treatment [17].

Acupuncture is a nonpharmaceutical therapy known to induce pain control, especially in the musculoskeletal system [18]. Various therapeutic effects of acupuncture are associated with central and systemic mechanisms involving the brain or autonomic nervous system as well as local effects at the acupuncture site [19]. Previous studies have shown that acupuncture changes the levels of neurotransmitters or hormones such as beta-endorphin, dopamine, serotonin, and cortisol [20,21,22] and has a specific effect on the limbic system or emotional area of the brain [19,23]. Also, other studies have further elucidated that acupuncture may be beneficial in treating chronic pain with depression [24], drug addiction [25], and degenerative disorders such as Alzheimer disease [26].

At present, with regard to potential advantages of acupuncture, case reports/series on acupuncture in athletes have been published steadily [27,28,29,30]. However, limited reviews with a comprehensive perspective on the use of acupuncture in athletes have been reported. Therefore, this systematic review aimed to summarize and analyze clinical case reports/series utilizing acupuncture for treating sports injuries of athletes, thereby providing the basis for further research to establish clinical evidence and expand the scope of acupuncture treatment in sports medicine. Furthermore, this study sought to provide useful insights for practitioners willing to utilize acupuncture in the clinical practice.

## 2. Materials and Methods

### 2.1. Search Strategy

To establish the search strategy that could comprehensively include clinical studies of athletes receiving acupuncture treatment for sports injuries, taking into account the search term sensitivity and specificity, a preliminary search was conducted in PubMed (https://pubmed.ncbi.nlm.nih.gov/) and domestic databases (Research Information Sharing Service (RISS, http://www.riss.kr/), National Digital Science Library (NDSL, http://www.ndsl.kr/), and Oriental Medicine Advanced Searching Integrate System (OASIS, https://oasis.kiom.re.kr/)). Based on these results, the following search terms were selected. The search terms used consisted of a combination of traditional Korean medicine treatment (“traditional Korean medicine”, “traditional Chinese medicine”, “acupuncture”, “herb*”, “moxibustion”, “cupping”, or “pharmacopuncture” (“aqua acupuncture”, “herb* acupuncture”, or “acupoint injection”)) and sports injury (((“wound”, “medicine”, or “injury”) and (“athletic” or “sport”)), “athletic injury”, “sports injury”, or “sports medicine”). Finally, a literature search was conducted in Embase (www.embase.com) including MEDLINE up to 21 August 2019 without language and publication date restrictions. Furthermore, the reference lists of related reviews were screened to ensure that there were no missing studies. We also manually identified additional records through other sources, including Google Scholar. All searched studies were listed and organized using Microsoft Excel (Microsoft, Redmond, WA, USA).

### 2.2. Study Selection, Data Extraction, and Data Analysis

The inclusion criteria were clinical case reports or series that studied the effects of treatment, including acupuncture, on sports injuries of athletes. To clarify this inclusion criterion, we reviewed and established several definitions among the authors. Firstly, we included manual acupuncture (MA), electroacupuncture (EA), auricular acupuncture, laser acupuncture (LA), acupressure, catgut embedding, dry needling, and transcutaneous electrical nerve stimulation (TENS) in the category of “acupuncture”. It is controversial whether TENS can be classified as a type of acupuncture; hence, we only included it in the analysis when the TENS was applied to the acupuncture points. Secondly, we defined case reports/series as a descriptive study based on uncontrolled observations containing detailed information on the diagnosis, treatment, response to treatment, and follow-up of individual patients. Several steps were taken based on the following exclusion criteria to select a study whose study design was a case report or a case series, regardless of the number of cases in an individual patient. The exclusion criteria were as follows: (1) Studies not targeted for humans (i.e., in vitro studies or animal studies), (2) research that did not collect original primary data (review, protocol, editorial, letter, etc.), (3) cross-sectional study or prevalence research, (4) experimental study with control (i.e., randomized controlled trials, controlled clinical trials), (5) no acupuncture-related treatment interventions (defined in advance) were used, (6) not targeted for athletes, (7) full text of the research results were not provided (i.e., conference poster abstract), and (8) studies not written in English, Chinese, or Korean. Since case reports/series are mostly retrospective observational studies, in many cases, different combination treatments are commonly used together, and it is ambiguous to distinguish which treatment was employed as the primary or adjuvant treatment. Therefore, we included all studies where the use of acupuncture as the main treatment was uncertain, but conducted a rigorous evaluation in the screening process to ensure that acupuncture was used for treatment in each individual study.

The records were screened accordingly and independently by two authors (J.-W.L. and S.-Y.K.) based on the title, abstract, or full text. First, all duplicate records belonging to the same title and author were removed. Grounded on the above inclusion/exclusion criteria, studies that were unrelated to sports medicine or studies that focused on doping, which fall into the category of sports medicine but failed to satisfy the inclusion criteria for this study, were first excluded for convenience. Next, the studies that corresponded to the exclusion criteria were removed to finally screen appropriate studies for our review analysis.

### 2.3. Data Extraction and Data Analysis

Data extraction was performed by one author (J.-W.L.) using predefined data extraction forms and then reviewed by another author (S.-Y.K.). If there was a discrepancy or further discussion was needed, it was resolved by another investigator (J.-H.L.), who was not involved in the extraction and review process. The data were extracted by classifying the following categories. These categories were modified based on some of the topic and checklist items in the CAse REport (CARE) guideline [31]: patient information, therapeutic intervention, follow-up, and outcomes were extracted. In the “patient information,” main concerns and symptoms of the patient including duration of the disorder, relevant past interventions, and patients’ demographic data such as type of sports, age, and gender were collected. For “therapeutic intervention”, types of acupuncture and acupuncture points, types of co-intervention, administration of intervention (such as treatment duration and number of treatment sessions), and changes in intervention with rationale were extracted. In relation to “follow-up and outcomes”, the outcome measures used in each study, their results, and AEs were collected, and information on RTP, recurrence, and conclusions from the authors of each study were summarized and analyzed.

The included sports were analyzed based on static components during competition according to the sports classification by Mitchell et al. [32]. If such sports did not quite match up to Mitchell et al.’s classification, we classified the sports in a consensus discussion between the authors.

Our study was not homogeneous enough to quantitatively synthesize the results of each individual study as not limited to a specific disease and did not include a control group. Therefore, it was not possible to evaluate the effectiveness by estimating the size of the effect or by statistically pooling and calculating the results between studies, and only descriptive and narrative results of qualitative analysis on the role of “acupuncture” described in each individual study were provided.

## 3. Results

**Figure 1:**
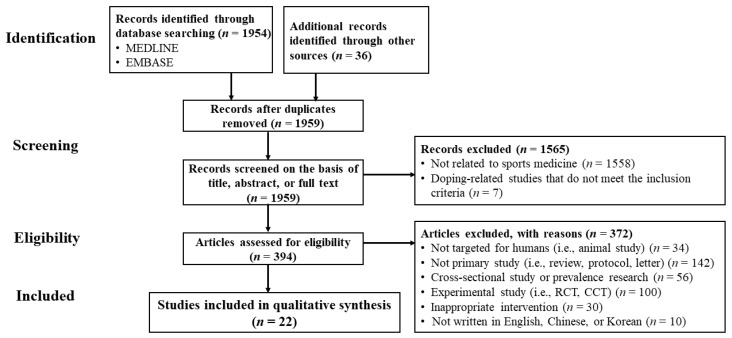
Flow diagram. CCT, controlled clinical trial; RCT, randomized controlled trial.

Our screening process is illustrated in Figure 1. Initially, 1954 potentially relevant articles were searched through the database, and 36 additional records were identified by manual searching. After removing duplicates (*n* = 31) from a total of 1990 records identified using the search strategy, 1959 studies were screened in view of the title, abstract, or full text, and 1937 were discarded (1558 studies were unrelated to sports medicine; 142 studies were not primary studies; 100 studies were experimental studies; 30 studies were not related to intervention using acupuncture points, etc.).

Finally, 22 case reports/series were included in our review [27,28,29,30,33,34,35,36,37,38,39,40,41,42,43,44,45,46,47,48,49,50]. The summary of the characteristics of the inclusion studies based on the items in the CARE checklist is shown in Table 1.

**Figure 2:**
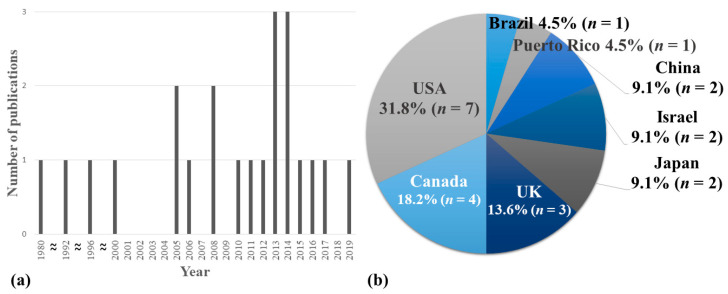
Characteristics of the included studies. (**a**) Number of published case reports/series by year; (**b**) frequency by country in which case reports/series were performed.

From 1980 to 2019, the number of relevant studies increased steadily with one study in the 1980s, two in the 1990s, six in the 2000s, and 13 in the 2010s. (Figure 2a). The studies were conducted in eight countries, with the largest number of studies in the United States (*n* = 7, 31.8%); followed by Canada (*n* = 4); the UK (*n* = 3); China, Israel, and Japan (*n* = 2, respectively); and Brazil and Puerto Rico (*n* = 1, respectively) (Figure 2b). Two studies conducted in China [40,41] were written in Chinese and the rest were written in English.

### 3.1. Demographic Information, Main Concerns, and Symptoms of the Patient

**Figure 3:**
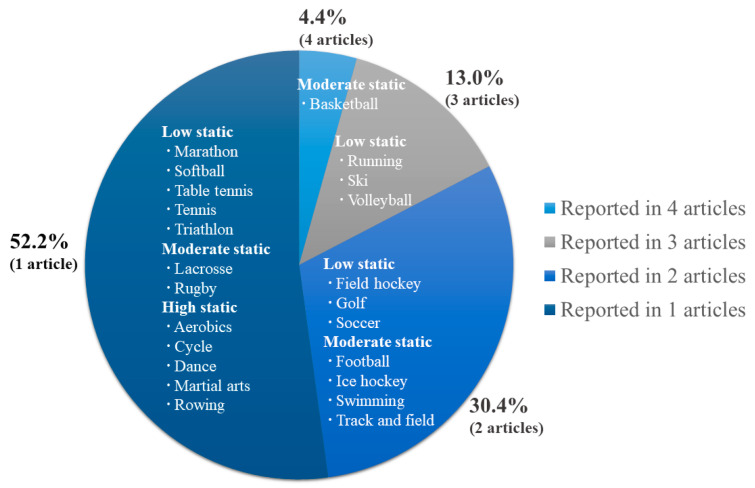
Type of sports. This figure shows how many case reports/series were performed according to the type of sports. For example, there were four case reports/series for basketball players, and three case reports/series for runners, skiers, and volleyball players, respectively. Each type of sports was classified and described according to the static components during competition.

All subjects included in this review were athletes, and sports injuries were reported in various sports such as basketball (*n* = 4, 9 cases); running, skiing, or volleyball (*n* = 3, respectively); and field hockey, golf, soccer, football, ice hockey, swimming, or track and field (*n* = 2, respectively). The details of each sport are shown in Figure 3. Most studies (*n* = 14) reported cases for one-sport type of athlete, but five studies included athletes of various types of sports, and three studies did not report any specific sport types.

The total number of athletes included in our review was 211, of which 119 (56.4%) were men. The athletes’ ages ranged from 8 to 77 years old (mean = 24.8).

**Figure 4:**
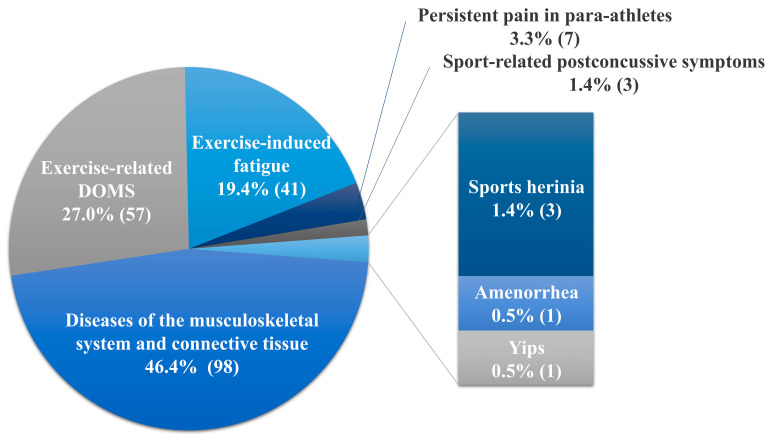
Main concerns and symptoms of the patients.

**Figure 5:**
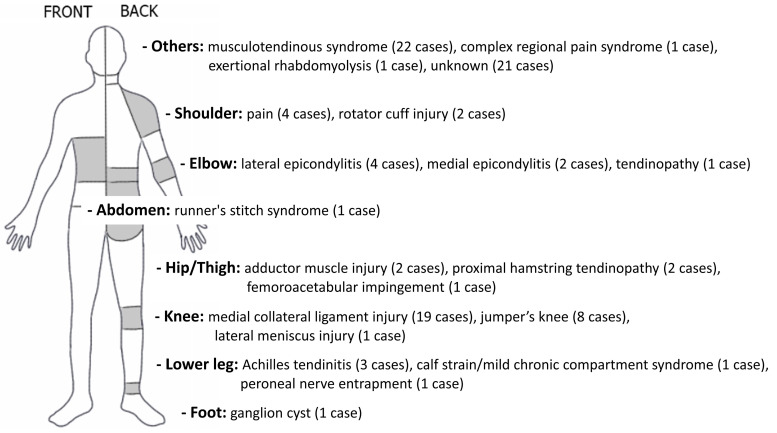
Injured body regions of musculoskeletal disease.

Figure 4 shows the main concerns and symptoms of athletes in the included studies. Of the 211 cases of sports injuries, musculoskeletal system and connective tissue diseases accounted for the highest proportion of primary symptoms reported in each case report/series (98 cases, *n* = 14 (46.4 %)). The most injured body regions were the knees (medial collateral ligament injury (19 cases), jumper’s knee (8 cases), lateral meniscus injury (1 case)), followed by the elbows (lateral epicondylitis (4 cases), medial epicondylitis (2 cases), tendinopathy (1 case)), and shoulders (pain (4 cases), rotator cuff injury (2 cases)). In addition, studies of diverse musculoskeletal disorders were reported, including musculotendinous syndrome with chronic pain in multiple body regions (22 cases), complex regional pain syndrome, exertional rhabdomyolysis, and ganglion cysts of the foot (1 case, respectively) (Figure 5). On the other hand, sports injuries other than the musculoskeletal system were as follows. There were two studies [29,30] on exercise-related delayed onset muscle soreness (DOMS, 57 cases) and one study [40] on exercise-induced fatigue (41 cases) in athletes. Two studies reported mental and behavioral disorders such as yips for golfers (1 case) [37] and postconcussive symptoms (3 cases) [28]. Other studies included persistent pain in para-athletes (7 cases) [48], sports hernias (3 cases) [44], and amenorrhea (1 case) [43].

The duration of the athletes’ disorder or symptoms varied from the day of onset to four years. According to relevant past interventions in each case report/series, among the patients of the included cases, except for seven studies, three cases had no past intervention, and all the other patients received conservative treatment such as physical therapy or medications for the symptoms. Of the seven studies excluded above, three studies conducted simply to observe the changes during the training periods did not require records for past intervention. The remaining four studies did not report relevant past interventions. The details are described in Table 1.

### 3.2. Therapeutic Intervention

#### 3.2.1. Acupuncture

Twelve of the 22 studies included in our review reported the use of MA at acupoints or myofascial trigger points (MTrPs). For EA, four studies used EA alone, while three studies used MA with EA. Other studies demonstrated catgut embedding, TENS at acupoints or MTrPs, or low-level LA at acupoints or ashi points (acupuncture site, not given a specific name or definite location, determined by tenderness or other pathological reactions).

Some trends were observed between the types of acupuncture and the category of diseases utilized. For example, MA with acupoints was used in various symptoms in our review, such as yips, amenorrhea, or sports hernia, in addition to musculoskeletal disorders. On the other hand, studies in which MA was applied only to MTrP, study that used EA alone without MA, TENS study, and LA study were mainly used for athletes with diseases of the musculoskeletal system and connective tissue.

The treatment sites were reported in detail as specific acupoints in 15 studies (Table 2). Among the remaining seven studies, six described treatment sites, not as particular acupoints, but as muscles or ranges. In addition, one study did not describe any treatment sites.

#### 3.2.2. Other Intervention (Co-Intervention)

In 12 studies that applied MA, only two studies that described golfer’s yips and runner’s stitch syndrome used MA alone, and all other studies used a variety of co-interventions with acupuncture. Moreover, excluding three studies applying TENS for musculotendinous syndromes, catgut embedding for exercise-induced fatigue, or EA for ganglion cysts of the foot, the other 10 studies used a combination of co-interventions with acupuncture. The types of co-intervention differed depending on the disease; most commonly used were medication, Tuina, and various physical therapies (Table 2).

#### 3.2.3. Treatment Duration and Session

The duration of treatment and follow-up periods for patients in each study varied from a few days to 13 months. With the exception of four studies that did not report the number of treatments, the study that provided the largest number of acupuncture treatments was the amenorrhea study in female runners (46 acupuncture treatment sessions over 13 months). Most of the others showed fewer than 10 treatment sessions.

Of the 11 studies reporting treatment frequency, the most common was a once- or twice-a-week regimen. Administration of treatment once a day or once every two weeks was reported in only one study, respectively.

Two studies showed that acupuncture was used only for some of the treatment periods: one study [50] used acupuncture in the second phase of the total three-phase treatment course, and the other study [35] used acupuncture only during the first two weeks.

### 3.3. Follow-Up and Outcomes

#### 3.3.1. Outcome Measures

The outcome measures used in the studies can be categorized as follows: (1) Subjective outcome measures, (2) objective outcome measures, (3) AEs, (4) information on RTP, and (5) recurrence rates, including follow-up results.

Many studies described narratively regarding the alteration or improvement of patients’ symptoms (*n* = 9). Different types of pain scales were used for an array of diseases that involve pain, including those of the musculoskeletal system, as follows (*n* = 7): Numerical rating scale (NRS), brief pain inventory (BPI), visual analog scale (VAS), pain relief score (PRS), McGill Pain Questionnaire (MPQ). There were also some objective outcome measures, such as blood test results, blood flow changes, body temperature, or numerical motor function evaluation outcome measures, including range of motion (ROM) (*n* = 6). Additionally, in some cases, results were comprehensively described on treatment days and cost for treatment, AEs, participants/providers ratio, effects on training, and recovery time, for the purpose of validity and feasibility evaluation of treatment intervention [29,30].

#### 3.3.2. Results

Most of the main outcomes focused on positive changes in patients. This can also be confirmed by the authors’ comments within each study on acupuncture treatment (Table 1). Of the 13 studies on the diseases of the musculoskeletal system and connective tissue, three studies used acupuncture only as an adjunctive treatment and acupuncture was not mentioned in the authors’ conclusions [39,41,46]. Several studies reported that MA played a significant role in the treatment of shoulder pain, elbow tendinopathy, proximal hamstring tendinopathy, or peroneal nerve entrapment. EA was highly recommended as a conservative intervention for the treatment of femoroacetabular impingement [27], lateral meniscus injury [35], and ganglion cysts of the foot [47]. According to Young [38], EA plus MA is very effective in the treatment of calf strain and tennis elbow. LA and TENS are effective in musculoskeletal disorders involving several regions.

In mental and behavioral disorders, MA for golfer’s yips and MA plus EA for sports-related postconcussive symptoms appeared to significantly reduce the symptoms. MA is beneficial and feasible for treating exercise-related DOMS in adolescent athletes and persistent pain in para-athletes. Catgut implantation at acupoints for sports fatigue or MA plus EA on soccer players with sports hernias appeared to significantly alleviate symptoms. As a result of Donoyama’s study [43], acupuncture or hormone replacement therapy for amenorrhea was not effective as a single intervention, but only when used in combination.

#### 3.3.3. Adverse Events

AEs were reported in five studies (22.7%), of which two reported no AEs, and the remaining three reported only minor AEs such as bleeding, pain, or numbness at the acupuncture site.

#### 3.3.4. Return to Play and Recurrence

Results of the patients’ RTP were reported in 12 studies (54.5%). In contrast, relatively less follow-up data related to relapse were collected, with only seven studies (31.8%) recording follow-up results to estimate recurrence rates (2-year follow-up period, *n* = 2; less than 6 months follow-up period, *n* = 5). As a result, only one study [33] reported that there was recurrence within the follow-up period; it was reported that six of 22 athletes treated for musculotendinous syndrome recurred between 10 days and two months after return to play.

## 4. Discussion

This systematic review summarized and analyzed case reports/series and series using acupuncture for sports injuries in athletes. Although the causal relationship of the effectiveness of acupuncture treatment was unclear due to the nature of the case report/series, it was confirmed that acupuncture was applied to a variety of sports injuries for athletes, ranging from yips of golf players to sports hernias of soccer players. The types of acupuncture also varied, including MA, EA, TENS, LA, and catgut embedding. The authors who performed each case report/series reported on the value of acupuncture as follows: (1) Short-term pain relief and recovery from dysfunction in each injured body region observed after acupuncture treatment could be beneficial in competitive elite athletes; (2) acupuncture has also been recognized for its clinical value as a useful noninvasive, conservative management for sports injuries such as lateral meniscus rupture, femoroacetabular impingement, ganglion cysts, and sports hernias; (3) acupuncture is worth trying for psychoneuromuscular impediments such as yips, which are poorly treated, and for exercise-related DOMS in athletes, and it has been suggested as an accessible treatment with appropriate resource (including cost and time).

Several previous RCTs have shown that acupuncture is effective in controlling pain in sports injuries in athletes, and it has been reported that the symptoms of rotator cuff tendinitis [51], shoulder impingement syndrome [52], shin splints [53], and plantar fasciitis [54] were improved by acupuncture. As of late, research on the effects and mechanisms of acupuncture tended to focus on pain control, but, clinically, it has been applied to various diseases and symptoms other than pain. Many studies related to pain control in acupuncture, which have been reported so far, have elucidated well the systemic and local mechanisms of acupuncture through mediators such as the central or autonomic nervous systems [19]. Nevertheless, research and discussion on the very complex elements involved in performing “acupuncture” are still in progress, and research on the specific mechanisms of the different diseases is still required.

As with the complexity of acupuncture, the scope of sports injuries is difficult and extensive to establish clearly. [55,56]. There are many different terms for sports injury, such as “sports impairment”, “sports trauma”, “sports incapacity”, “sports disease”, and “sports illness”. If it can be recognized that each of these terms may have subtle differences in meaning, it can be seen that the need for a very complex perspective and a multidisciplinary approach in the field of sports medicine is inevitable. In fact, sports injury is known to require a systematic and multidisciplinary approach to treatment due to its complex etiology and various symptoms [7]. For example, overuse injuries in athletes are difficult to treat because symptoms are often extensive and uncharacteristic [57]. Another factor to consider in the treatment of sports injury in athletes is that they need not only recovery of the injured body part, but also recovery of their function to perform the sport again [15]. For athletes to return to play, systematic rehabilitation is necessary following successful treatment [58]. Physical and muscle strength, muscular endurance, flexibility, and agility need to be improved again as well as management of anxiety and depression caused by injury, if necessary [17]. Therefore, for sports injuries of elite athletes, an “RTP strategy” is required, and, for this, it has been discussed that the conservative method using a multimodal approach should be considered first [27]. However, apart from the lack of evidence of recommendations for various conservative treatment options [59], there are still limitations in the application of drug therapy, one of the most used conservative managements, to all cases of the RTP strategy [60]. The case studies included in this review suggest that acupuncture is one of the conservative approaches that would merit consideration in the field of sports and may be worth trying even for unexpected disorders that are not easy to treat [27,28,29,30,33,34,35,36,37,38,39,40,41,42,43,44,45,46,47,48,49,50].

Interestingly, case reports/series included in this review were mainly performed in the Americas and Europe, but not in the East Asian countries where acupuncture is primarily used in clinical practice. However, this does not denote that acupuncture treatments are not applied to sports injuries in East Asian countries. In Korea, acupuncture has been used formally or informally in sporting events since the early 1990s. In international sports competitions held in Korea that encompass a variety of sports, team doctors treated Korean athletes (sometimes including foreign athletes) with acupuncture and expressed satisfaction [61,62,63]. This positive experience has led to an opportunity for acupuncture to be introduced into the official medical treatment of the Olympic Village polyclinic in the 2018 PyeongChang Winter Olympics [64].

For a critical interpretation of this review, we suggest that careful consideration of the following limitations is essential. Firstly, the distinctive advantage of this review, but also an intricate disadvantage, was that we analyzed “sports injury” research on “athletes”. Defining the terms “sports injury” and “athlete” involves a multitude of controversial concepts. [65] Thus, future research suggesting an accurate terminology will provide a clearer perspective. Second, it should be taken into account that the data we analyzed were derived from case reports/series. For our research to provide useful insights for clinical acupuncture for sports medicine practitioners, it is necessary to establish specific recommendations for which acupuncture is an effective treatment for the different types of sports injuries. This cannot be presented based only on case reports/series. The third limitation is linked to the second limitation. It should be noted that acupuncture treatment may not be a “study only to verify the effectiveness of acupuncture” in each case study. Since a number of studies included in this review used acupuncture as a co-intervention, or an adjunctive treatment, and not a stand-alone intervention, the effect of acupuncture can be a response to a multimodal conservative treatment. Fourth, our research showed that acupuncture can be applied to a variety of diseases within the sports injury category in athletes, but should not be misleading in the sense that acupuncture is effective for all variety of diseases. Experimental research such as RCTs for athletes, or systematic review based on it, can give a clear answer to the effectiveness of acupuncture. A recent systematic review of the effects of acupuncture treatment on DOMS reported that acupuncture had small-to-moderate effects compared to the no-treatment group and presented insufficient data for meta-analysis [66]. Further well-designed studies assessing acupuncture’s efficacy in sports medicine are warranted to establish a strong evidence and to support clinical practice.

This study provided minor clues about the possibility of applying acupuncture to various types of sports injuries in athletes. In the future, we need to clarify which types of injuries are more effective in acupuncture through a detailed classification of sports injuries. As previously stated, it is very difficult to clearly identify the causes or symptoms of sports injuries. Nevertheless, in order to propose a more practical application of acupuncture, research that can generate evidence through more specific research questions and hypotheses should be conducted.

## 5. Conclusions

In this review, we endeavored to identify the potential of acupuncture for managing discomfort in athletes and their return to exercise through case reports/series on sports injuries in athletes. The included cases showed some potential of acupuncture in the treatment of various types of sports injuries, beyond pain control in musculoskeletal disorders. In addition, this review will assist practitioners who wish to use acupuncture to treat sports injuries and enable practical clinical applications of acupuncture, thereby allowing the use of novel therapeutic options for patients. This is the first review, to our knowledge, to systematically search, summarize, and analyze clinical case reports/series on the potential application of acupuncture in the treatment of sports injuries of athletes.

## Figures and Tables

**Table 1:** Summary of included case reports/series.

Study			Demographic Information			Main Concerns and Symptoms of the Patient		Medical History	Administration of Intervention	Follow-up and Outcomes				
No	Study ID	Coun. (Lang)	Sports type	Age (yr)	No. of Cases	Disorders	Duration	Previous Interventions	① Interventions ② Duration ③ No. of sessions	Outcome measures	Results	Adverse events	① Return to play ② Recurrence (f/u)	Authors’ comments for acupuncture treatments
Diseases of the musculoskeletal system and connective tissue														
1	Dlin, 1980 [33]	Israel (Eng.)	ND	12–44 (mean = 2(5)	22	Musculotendinous syndromes of the chronic exertion type (shoulder, knee, anterior tibia, back pain)	Few wks-several mon	At least a 2 to 3 wk period: [all patients] heat application, rest; [as needed] oral anti-inflammatory medication (indomethacin), physiotherapy (ultrasound, or local steroid injections)	① TENS ② ND ③ 2–6	(1) Pain questionnaire (2) Pain grade	(1) Complete pain relief (*n* = 1(5), good (>50% reduction) (*n* = (4), fair (30–50% reduction) (*n* = (1), poor (<30% reduction) (*n* = (2) (2) Grade 0 (*n* = 17), 2 grades reduction (good result) (*n* = (2), 1 grade reduction (fair result) (*n* = (1), No changes (*n* = (2)	None	① 18 of 22 subjects returned to full sports activity ② 10-day-2-mon after return to full sports activity (*n* = 6)	*TENS stimulation of acupuncture points may be effective in the therapy of painful sports injuries of the athletic exertion type.*
2	Sternfeld, 1992 [34]	Israel (Eng.)	Long-distance running	16	1	Runner’s stitch syndrome	Several mon	Analgesics and NSAIDs	① MA ② 31-day [1/day (3-day)+1/wk (4-wk)] ③ 7	Symptoms	Complete and permanent recovery	ND	① Resuming all activities and achievements in competitive sports ② None (several wks)	*We suggest acupuncture as a preferred therapy for cases involving runner’s stitch syndrome.*
3	Seplow, 1996 [35]	USA (Eng.)	Basketball	17	1	Lateral meniscus injury (a tear of the posterior horn of the lateral meniscus)	3-day	Ice, rest, NSAIDs (2-day)	① EA + others ② Initial 2-wk ③ ND	Symptoms	Walk without limp or pain and range of knee flexion returned to 130°	ND	① Return to training in 5-wks, full participation in 8-wks ② ND	*When tears are diagnosed, conservative methods may be effective in treating these athletes and may in fact be more effective in many cases.*
4	Hoven, 2000 [36]	USA (Eng.)	Ski	19	1	Peroneal nerve entrapment and ankle sprain	ND	Pelvic/lower extremity chiropractic manipulation, interferential current, cryotherapy (3-day)	① MA + others ② 5-day ③ 5	Symptoms	Pain and weakness alleviated after 3-day of care	ND	①, ② ND	*It appears that chiropractic manipulation of the lumbar, pelvis and lower extremity, combined with interferential current, cryotherapy, acupuncture and trigger point therapy played a significant role in alleviating the peroneal neuropathy experienced by this elite junior national skier.*
5	Young, 2005 [38]	UK (Eng.)	Rugby	#1: 19; #2: 32	2	#1: Calf strain/mild chronic compartment syndrome; #2: Tennis elbow/muscle tightness	#1: 6-mon; #2: ND	ND	① EA + MA + others ② #1: 6-wk #2: 4-wk (2/wk) ③ #1: 12; #2: 8	Symptoms	#1: Completion of the full training without pain #2: Training at ease	ND	① #1: Playing games without pain; #2: Playing at ease ② ND	*Even though using acupuncture is very effective in treatment, there comes a time in the treatment of an injury when you will need to open the channels physically to allow qi and blood to flow smoothly and to return the shortened muscle back to its normal length.*
6	Martínez-Silvestrini, 2006 [39]	Puerto Rico (Eng.)	ND	11	1 *	CRPS after lower extremity trauma and clinical depression	Few wks	(For CRPS treatment) Hydrotherapy, electrical stimulation, ultrasound to the Achilles tendon, stretching, active assisted range of motion, weight bearing, opioid analgesics, corticosteroids	① MA + others ② 3-mon ③ ND	Symptoms	The clinics discharge without any deficits	ND	①, ② ND	*ND*
7	Yan, 2008 [41]	China (Chin.)	Football	Mean = 21	19 **	Sports injury in knee joint medial collateral ligament	ND	ND	① EA + others ② ND (one course comprised 7-day) ③ ND	(1) Recovery time (2) Therapeutic effect (3) The overall efficiency	(1) Rapid (23%); moderate (50%) (2) Recovered (40%); partly recovered (53%) (3) 93%	ND	①, ② ND	*ND*
8	Osborne, 2010 [42]	UK (Eng.)	Volleyball	23–27 (mean = 2(5)	4	Anterior/anterolateral shoulder pain ^(1)^	#1: 2 mon.; #2: 6 yr.; #3: 18 mon.; #4: 6-mon	Conservative treatment (#1: Thera band, stretching; #2, #3: ND; #4: exercises, stretching)	① MA + others ② 1-mon-long intense competitive phase ③ #1, #3, #4: 1; #2: 2	(1) Functional assessment (MPQ) (2) ROM (3) Verbal pain scores (4) Muscle strength	(1)-(4) All scores improved	ND	① Continuing overhead activities ② ND	*These cases support the use of dry needling in elite athletes during a competitive phase with short-term pain relief and improved function in shoulder injuries.*
9	Pearcey, 2013 [46]	Canada (Eng.)	ND	31	1	Exertional rhabdomyolysis	Few days	Intravenous hydration with sodium bicarbonate and blood work [3 days]	① MA + others ② 2 day ③ ND	CK levels	Declined by 45%	ND	① 1 mon. after the diagnosis, the subject slowly returned to high-intensity resistance training (>2/wk.) without any complications ② ND	*ND*
10	Woitzik, 2013 [47]	Canada (Eng.)	Cycle	45	1	Ganglion cyst of the foot	10-day	ND	① EA ② 4-wk (1/wk) ③ 4	Symptoms	Resolution of the cyst (11 day after final treatment)	ND	①, ② ND	*EA may be a novel and non-invasive conservative approach for the treatment of ganglion cysts.*
11	Morimoto, 2013 [45]	Japan (Eng.)	Various sports ^(2)^	17–77 (mean = 39)	41	Jumper’s knee, lateral/medial epicondylitis, Achilles tendinitis, adductor muscle injury, rotator cuff injuries, etc.^(3)^	ND	ND	① LA + others ② ND ③ 2–10 (mean = 4.(1)	Pain Relief Score	5 or less (*n* = 27,66%)	ND	①, ② ND	*Low-level LA is an effective treatment for sports injuries, particularly jumper’s knee, tennis elbow and Achilles tendinitis.*
12	Gliedt, 2014 [49]	USA (Eng.)	Basketball	41	1	Sub-acute left elbow tendinopathy	5 wk	Self-massage, OTC NSAIDs	① MA + others ② ND (1/wk) ③ 2	Symptoms	Reduced left elbow swelling and left wrist extensor muscle hypertonicity, and no pain	ND	① A return to normal recreational athletic activities including basketball ② None (6-wk)	*The patient’s outcomes indicated a quick resolution of subjective complaints and objective findings with the chosen treatment.*
13	Jayaseelan, 2014 [50]	USA (Eng.)	Running	#1: 70; #2: 69	2	Proximal hamstring tendinopathy	#1: 7 mon; #2: 5 mon	Eccentric training of hamstrings, lumbopelvic stabilization exercises, patient education (2-wk: “Phase 1” treatment)	① MA + others ② #1: 3 wk; #2: 4 wk (“Phase 2” treatment) (1/wk) ③ 3	(1) Pain (NRS) (2) Global rating of change (3) Lower Extremity Functional Scale (4) Tenderness (5) Strength	(1)–(5) All score improvement, significant function improvement	ND	① Each patient returned to running and sitting without symptoms ② None (6 mon)	*In these 2 runners, eccentric loading of the hamstrings, lumbopelvic stabilization exercises, and trigger point dry needling provided short- and long-term pain reduction and functional benefits.*
14	MacIntyre, 2015 [27]	Canada (Eng.)	Ice hockey	22	1	FAI	4 yr	None	① EA + others ② 6-wk (1–2/wk) ③ 8	Symptoms	No pain [at rest, daily activities (including exercise), all stress tests used for initial physical examination]; despite having less pain, the hip scour and FADIR tests still provided a hard end-feel and palpable click at extreme ROM	ND	① At 8 wk ② None (6 mon)	*Conservative management utilizing a multimodal approach, as described in the case, should be first line treatment.*
Exercise-induced fatigue														
15	Chen, 2008 [40]	China (Chin.)	Rowing, track and field, swimming, table tennis ^(4)^	15–26 (mean = 20)	41	Exercise-induced fatigue	N/A	N/A	① Catgut embedding ② 90–120 day ③ 6	(1) Training-related condition (2) Changes level of blood testosterone and hemoglobin	(1) Significantly improved (2) Significantly increased	ND	①, ② N/A	*Catgut implantation at acupoints can significantly improve exercise fatigue in the player and the mechanism is possibly related with increase of testosterone and hemoglobin levels.*
16	Garlanger, 2017 [29]	USA (Eng.)	Nordic ski	14–17	15	Exercise-related DOMS and sense of well-being	N/A	N/A	① MA + others ② 2 wk ③ 1–5	(1) Time required by research staff on treatment days (2) Cost (3) AEs (4) Participant/provider ratio (5) Effect on DOMS 6) Effect on sense of well-being	(1) 90 min (2) 1500 USD (3) (See next column) (4) 7:1 (5) significantly improved (*p* < 0.05) 6) NS	No severe AEs; Minor AEs (73%, site pain, etc.)	①, ② N/A	*Providing acupuncture to adolescent Nordic ski athletes in the practice field under extreme temperature is feasible with the appropriate resources.*
17	Luetmer, 2019 [30]	USA (Eng.)	Football	13–18 (mean = 16)	42	Exercise-related DOMS and sense of well-being	N/A	N/A	① MA + others ② 5-day ③ 5 (*n* = 1(1); 4 (*n* = 1(3); 3 (*n* = 8); 2 (*n* = 6); 1 (*n* = (4)	Same as above	(1) 75 min (2) 700 USD (3) (See next column) (4) 7–10:1 (5) significantly improved (*p* < 0.001) (6) NS	No severe AEs; Minor AEs (55%, mild focal numbness or tingling)	①, ② N/A	*Effectively providing acupuncture to multiple adolescent football players in their training environment is feasible with appropriate staff and resources.*
Others														
18	Abe, 2014 [48]	Brazil (Eng.)	Various sports (Paralympic athletes) ^(5)^	Mean = 35	7	Persistent pain (shoulder, elbow, spine, or knee) ^(6)^	#1: 4-wk; #2-#4, #6: chronic, intermittent; #5: 2-wk; #7: 8-wk	Oral/topical NSAIDs, muscle relaxant, physiotherapy, immobilization ^(7)^	① MA + others ② 6 wk (2/wk) ③ 12	(1) Pain (VAS) (2) Pain (MPQ)	(1), (2) Significant pain reduction	No severe AEs; Minor AEs (episodic local bleeding); One athlete died of liver cancer after this study	①, ② ND	*Pain symptoms were reduced with acupuncture. The mean duration required for improvement was eight acupuncture sessions.*
19	Lin, 2016 [28]	USA (Eng.)	#1: Ice hockey #2: Field hockey and lacrosse #3: Soccer	#1: 8; #2: 15; #3: 18	3	Sport-related postconcussive symptoms	#1,#2: 1 mon; #3: 1.5 mon	Physical therapy, medications ^(8)^	① MA + EA + others ② #1, #2, 6-wk; #3, 14-wk (1/wk) ③ #1, #2, 6; #3, 14	(1) Pain (VAS) (2) Pain (BPI) (3) Postconcussive symptoms questionnaire	(1)-(3) Satisfactory symptomatic reduction in all patients	None	① #1, #2: ND; #3: Return to all sports activities (All patients returned to their physical, social, and school activities) ② ND	*Acupuncture with conventional medication appeared to reduce the postconcussive symptoms in the 3 patients.*
20	Yuill, 2012 [44]	Canada (Eng.)	#1: Soccer #2, #3: Soccer and hockey	#1: 23; #2: 18; #3: 29	3	Sports hernia (chronic groin pain)	#1: 5 wk; #2: 3-mon; #3: 5-wk	#1: None; #2: Ice, interferential current, ultrasound (2 mon); #3: Physiotherapy, adductor stretching/strengthening, interferential current, ultrasound	① MA + EA + others ② 6–8-wk (1–2/wk) ③ 6–16	(1) Pain (VAS) (2) Resisted muscle testing	(1) 0/10 in all cases at the time of patient discharge (2) All positive tests changed to negative	ND	① Within 3–4 days of last therapy ② None (2 yr)	*Three soccer players, of varying levels of ability, presenting with a suspected sports hernia were relieved of their pain after 8 weeks of conservative care.*
21	Donoyama, 2011 [43]	Japan (Eng.)	Middle- and long- distance running	26	1	Amenorrhoea	Several mon	A norgestrel-ethinyl estradiol combination (2-wk]	① MA + others ② 13 mon ③ 46	(1) Menstrual blood flow (2) Basal Body Temperature (3) Body Weight (4) Body Fat Rate (5) BMI	(1) Amount and duration of menstrual flow increased at regular intervals (2) Moved close to the biphasic pattern (3)-(5) NS	ND	①, ② ND	*Acupuncture treatment may be a feasible treatment option in the field of sports medicine to help competitive athletes with amenorrhoea.*
22	Rosted, 2005 [37]	UK (Eng.)	Golf	65	1	Yips	2 yr	None	① MA ② Few weeks (1/wk+1/2-wk) ③ 8	Symptoms	Symptoms disappeared after 1 treatment	ND	① Playing golf using his right hand without symptoms ② None (2 yr)	*Acupuncture may be worth trying in patients with the yips since this condition is otherwise difficult to treat.*

AE, adverse event; BMI, body mass index; BPI, brief pain inventory; Coun, country; Chin., Chinese; CRPS, complex regional pain syndrome; DOMS, delayed onset muscle soreness; EA, electroacupuncture; Eng., English; FADIR test, flexion-adduction-internal rotation test; FAI, Femoroacetabular impingement; f/u, follow-up; L, left; LA, laser acupuncture; lang, language; MA, manual acupuncture; min, minutes; mon, month; MPQ, McGill pain questionnaire; N/A, not applicable; ND, not described; NRS, numeric rating scale; NSAID, nonsteroidal anti-inflammatory drug; OTC, over-the-counter; R, right; ROM, range of motion; SLAP, superior labrum tear from anterior to posterior; TENS, transcutaneous electrical nerve stimulation; VAS, visual analogue scale; wk, week; yr, year. *Of all three cases, one was treated with acupuncture. ** Of all 30 cases, 19 were treated with acupuncture. ^(1)^ #1: Intermittent discomfort. #2: Partial nonrepaired SLAP lesion but with no functional impact. #3: Right arthroscopic capsular tightening, intermittent pain. #4: Intermittent pain in right shoulder. ^(2)^ Golf (*n* = 6), martial arts (*n* = 4), basketball (*n* = 4), marathon (*n* = 3), aerobics (*n* = 2), ski (*n* = 2), softball (*n* = 2), dance (*n* = 2), tennis (*n* = 2), triathlon (*n* = 2), volleyball (*n* = 2), etc. (*n* = 10). ^(3)^ Jumper’s knee (*n* = 8), lateral epicondylitis of humerus (*n* = 3), Achilles tendinitis (*n* = 3), adductor muscle injury (*n* = 2), medial epicondylitis of humerus (*n* = 2), rotator cuff injuries (*n* = 2), etc. (*n* = 21). ^(4)^ Rowing (*n* = 7), track and field (*n* = 20), Swimming (*n* = 12), table tennis (*n* = 2); ^(5)^ #1, #4-#6: Basketball #2: Swimming and volleyball. #3: Swimming. #7: Track and field (paralympic athletes). ^(6)^ #1: Right shoulder and Right elbow. #2: Spine. #3: Left knee, shoulders, and spine. #4: Left shoulder. #5: Shoulders and spine. #6: Shoulders. #7: Left shoulder. ^(7)^ #1: Oral and topical NSAIDs (three days after onset). #2, #3: NSAIDs and physiotherapy (immediately after onset). #4, #6: NSAIDs (immediately after onset); #5: muscle relaxant and NSAIDs, physiotherapy (cryotherapy), immobilization (immediately after onset). #7: NSAIDs (four weeks; intermittent); ^(8)^ #1: Physical therapy and medications (acetaminophen, ibuprofen, or amitriptyline) (20 mg/day). #2: Acetaminophen (every day), ibuprofen (only for severe headaches), amitriptyline (20 mg/day), physical therapy, vestibular therapy; #3: Ibuprofen (as needed, ~2/wk), physical therapy (2/wk).

**Table 2:** Details of acupuncture and co-intervention.

No.	Study ID	Sports Type	Disorders	Details of Acupuncture (Acupuncture Sites)	Co-Intervention
Diseases of the musculoskeletal system and connective tissue					
1	Dlin, 1980 [33]	ND	Musculotendinous syndromes of the chronic exertion type (shoulder, knee, anterior tibia, and back pain)	TENS (MTrPs or acupoints)	None
2	Sternfeld, 1992 [34]	Long-distance running	Runner’s stitch syndrome	MA (PC3, LR14, ST36, SP9, LR2, SP3)	None
3	Seplow, 1996 [35]	Basketball	Lateral meniscus injury (a tear of the posterior horn of the lateral meniscus)	EA (ST41, GB43, BL54, BL60)	Ice, kinesiology, resistance bands
4	Hoven, 2000 [36]	Ski	Peroneal nerve entrapment and ankle sprain	MA (ST40, GB34, GB35, GB36)	Chiropractic manipulation (lumbar spine, pelvis, lower extremity), interferential current, cryotherapy, soft tissue mobilization
5	Young, 2005 [38]	Rugby	#1: Calf strain and mild chronic compartment syndrome #2: Tennis elbow and muscle tightness	#1: - EA (BL55-BL57, BL57-GB35) - MA (BL40, BL57, BL58, BL60, BL65, GB34, GB41, LI4) #2: - EA (ashi points, EX-B2 from C3 to L5) - MA (ashi points, GB21, LI15, TE14, TE5, LI10, LI11, LI12, LI5, LI4, LI3, TE3)	#1: Tuina (gun-fa, rolling technique; rou-fa, kneading; plucking (frictions)), stretching, patient education #2: Tuina (gun-fa), cupping (epicondyle)
6	Martínez-Silvestrini, 2006 [39]	ND	CRPS after lower extremity trauma and clinical depression	(for clinical depression and pain disorder treatment) MA (ND)	(for clinical depression and pain disorder treatment) Fluoxetine (SSRIs)
7	Yan, 2008 [41]	Football	Sports injury in knee joint medial collateral ligament	EA (EX-LE2, EX-LE5, GB34)	Massage, physiotherapy, cold pack, electric stimulation, external preparation, surgery, splint, rehabilitation training (as needed)
8	Osborne, 2010 [42]	Volleyball	Anterior/anterolateral shoulder pain	MA (MTrPs: scapulohumeral muscles)	Soft tissue therapy around the shoulder, post-training icing strategies, exercises and stretching
9	Pearcey, 2013 [46]	ND	Exertional rhabdomyolysis	MA (from shoulder to hand)	Intravenous hydration with sodium bicarbonate and blood work
10	Woitzik, 2013 [47]	Cycle	Ganglion cyst of the foot	EA (ST36, LR3, 2 needles inserted into the cyst on opposite sides stimulated at 5 Hz)	None
11	Morimoto, 2013 [45]	Various sports	Jumper’s knee, lateral epicondylitis of humerus, Achilles tendinitis, adductor muscle injury, medial epicondylitis of humerus, rotator cuff injuries, etc	LA (low level laser therapy at points of pain/or acupuncture points)	Medication (NSAIDs) or a poultice (as needed)
12	Gliedt, 2014 [49]	Basketball	Sub-acute left elbow tendinopathy	MA (palpated areas of tenderness, just distal to the insertion site of the common wrist extensor muscles)	HVLA spinal manipulation (thoracic spine), ART (elbow), home exercise program (Brugger’s exercises for postural dysfunction)
13	Jayaseelan, 2014 [50]	Running	Proximal hamstring tendinopathy	MA (MTrPs: medial/lateral hamstrings, adductor magnus muscle)	Eccentric training of hamstrings, lumbopelvic stabilization exercises, patient education
14	MacIntyre, 2015 [27]	Ice hockey	FAI	EA (2 Hz, L2-L5 bilaterally, and hip muscles and nerves, SP12, LR10, GB29, BL53, BL54)	Soft tissue therapy, spinal manipulative therapy, MWM and rehabilitation exercises
Exercise-induced fatigue					
15	Chen, 2008 [40]	Rowing, track and field, swimming, table tennis	Exercise-induced fatigue	Catgut embedding (CV4, BL23, GV4, ST36, SP6 and etc.)	None
16	Garlanger, 2017 [29]	Nordic ski	Exercise-related DOMS and sense of well-being	MA (when underlying muscle soreness occurred: SP10, ST34, SP9, ST36, GB34, BL56; if no specific muscle soreness: ST36, GB34, ST34)	NSAIDs (24–48 h prior to the study, *n* = 6; between treatment days 2 and 3, *n* = 3; between treatment days 4 and 5, *n* = 1)
17	Luetmer, 2019 [30]	Football	Exercise-related DOMS and sense of well-being	MA (when underlying muscle soreness occurred: SP10, ST34, SP9, ST36, GB34, BL56; if no specific muscle soreness: ST36, GB34, ST34)	NSAIDs or acetaminophen (before day 1, *n* = 7; between day 1 and 2, *n* = 7; between days 2 and 3, *n* = 10; between days 3 and 4, *n* = 8; between days 4 and 5, *n* = 9)
Others					
18	Abe, 2014 [48]	Various sports (Paralympic athletes)	Persistent pain (shoulder, elbow, spine, or knee)	MA (CV12, CV3, LI4, LU7, HT7, SP10, ST36, LR2, KI3)	The training schedule and the usual treatment regimens maintained
19	Lin, 2016 [28]	Ice hockey, field hockey, lacrosse, soccer	Sport-related postconcussive symptoms	- MA (#1: LI4, ST36, LR3, TE5, Ashi points; #2: LI4, ST36, LR3, TE5, SP6, Yin-Tang, Ashi points; #3: LI4, ST36, SP6, LR3, TE23, GV20, Yin-Tang, TE5, LI11, ST7, BL4, BL6, TE9, GB21, GB20, Ashi points) - EA(2-Hz, ST 36)	Cupping (Back Shu points), Gua sha (Back Shu points, treatment involving repeated pressured strokes over body surface with a smooth edged tool), conventional medical therapies maintained
20	Yuill, 2012 [44]	Soccer, hockey	Sports hernia (chronic groin pain)	- EA (2 Hz, L2-L4, T10-T12 bilaterally) - MA (#1: SP 13, ST 29, ST30, KI11, GB28 #2: ST29, ST30, LR9, LR10 #3: ST30, LR10)	Soft tissue therapy, laser therapy and microcurrent (site of the chief complaint), stretching, Wobenzyme (2400mg tabs, 2/day, 2 weeks), rehabilitation therapy, plyometric training (3/week, 8 weeks)
21	Donoyama, 2011 [43]	Middle- and long- distance running	Amenorrhoea	MA (CV6, CV12, LR3, LR14, BL17, BL18, BL23, SP6, SP10)	A norgestrel-ethinyl estradiol combination (as needed)
22	Rosted, 2005 [37]	Golf	Yips	MA (GV20, EX-HN-1, TE5)	None

ART, active release techniques; BL, bladder meridian; CRPS, Complex regional pain syndrome; CV, conception vessel meridian; DOMS, Delayed onset muscle soreness; EA, electroacupuncture; EX-B, extra point (back); EX-HN, extra point (head and neck); EX-LE, extra point (lower extremity); FAI, Femoroacetabular impingement; GB, gallbladder meridian; GV, governor vessel meridian; HT, heart meridian; HVLA, high-velocity low-amplitude; KI, kidney meridian; LA, laser acupuncture; LI, large intestine meridian; LR, liver meridian; LU, lung meridian; MA, manual acupuncture; MTrPs, myofascial trigger points; MWM, Mulligan Mobilizations with Movement; ND, not described; NSAID, nonsteroidal anti-inflammatory drug; PC, pericardium meridian; SP, spleen meridian; SSRIs, selective serotonin reuptake inhibitors; ST, stomach meridian; TE, triple energizer meridian; TENS, transcutaneous electrical nerve stimulation.
